# Nanocarriers Provide Sustained Antifungal Activity for Amphotericin B and Miltefosine in the Topical Treatment of Murine Vaginal Candidiasis

**DOI:** 10.3389/fmicb.2019.02976

**Published:** 2020-01-10

**Authors:** Fernanda Walt Mendes da Silva de Bastiani, Cristina de Castro Spadari, Jenyffer Kelly Rocha de Matos, Giovanna Cassone Salata, Luciana Biagini Lopes, Kelly Ishida

**Affiliations:** ^1^Laboratory of Antifungal Chemotherapy, Department of Microbiology, Institute of Biomedical Sciences, University of São Paulo, São Paulo, Brazil; ^2^Laboratory of Nanomedicine and Drug Delivery Systems, Department of Pharmacology, Institute of Biomedical Sciences, University of São Paulo, São Paulo, Brazil

**Keywords:** antifungal, alginate nanoparticles, microemulsions, drug delivery, vaginal candidiasis, *Candida*

## Abstract

Topical drug administration is frequently used for the treatment of vaginal candidiasis; however, most formulations using this route do not provide prolonged drug release. Our aim was to evaluate the antifungal efficacy of amphotericin B (AMB) and miltefosine (MFS) incorporated in nanocarriers for sustained drug release, in a murine model of vaginal candidiasis. AMB and MFS were incorporated in different topical formulations, namely: conventional vaginal cream (daily dose for 6 days; MFS-CR and AMB-CR groups), microemulsion that transforms into a liquid crystalline gel *in situ* (single dose, or in three doses, every 48 h; AMB-ME and MFS-ME groups) and alginate nanoparticles (single dose; MFS-AN group). Formulations were administered intravaginally in BALB/c female mice 24 h post-infection by *Candida albicans* yeasts. On the 7th day post-infection the animals were euthanized for mycological and histological analyses. Formulation persistence in the vaginal canal was assessed for 7 days by *in vivo* imaging, using nanocarriers labeled with Alexa-Fluor 647. AMB-ME(3×), MFS-ME(3×), and MFS-AN(1×) formulations were able to control fungal infection at comparable levels to those vaginal cream formulations. Notably, a single dose of MFS-AN was sufficient to reduce the fungal burden as effectively as MFS-ME(3×) and MFS-CR(6×). *In vivo* imaging showed that nanocarriers allowed prolonged antifungal activity by intravaginal administration reducing drug administration frequency. Therefore, AMB and MFS incorporated into a microemulsion and MFS encapsulated in alginate nanoparticles could be effective therapeutic alternatives for vaginal candidiasis, likely due to the sustained antifungal activity provided by these nanocarriers.

## Introduction

Vulvovaginal candidiasis (VVC) is one of the most frequent diseases of the female genital tract at reproductive age, affecting 75% of women at least once in their life time (De Bernardis et al., 2018). Approximately 5% of VVC cases develop into a severe form of the disease known as recurrent vulvovaginal candidiasis (RVVC), which is characterized by more than three episodes in a one-year period (Denning et al., 2018). *Candida albicans* is the most frequent species in VCC and RVVC cases, although fluconazole-resistant and *Candida* non-*albicans* strains (CNA, eg., *C. glabrata*) are also associated with RVVC (Dovnik et al., 2015; Denning et al., 2018).

The treatment of VVC can be performed using topical drug formulations, most frequently vaginal creams containing imidazole, triazole or nystatin, which are administered once a day for up to 14 days (Pappas et al., 2015). In contrast, RVVC requires longer antifungal therapy using topical formulations administered for up to 6 months (Pappas et al., 2015); alternatively, oral administration with fluconazole or itraconazole for up to 6 months may be required (Pappas et al., 2015). As an alternative for RVVC treatment, amphotericin B (AMB) intravaginal suppository or vaginal cream are used due to their strong fungicidal effect and broad antifungal spectrum; in addition, resistance to AMB is rare (Feuerschuette et al., 2010; Ci et al., 2018).

Previous studies demonstrated that miltefosine (MFS) has fungicidal effect and a broad-spectrum of activity, including against fluconazole-resistant *Candida* spp. isolates, demonstrating that it is a potential drug for the treatment and/or prevention of fungal infections (Widmer et al., 2006; Dorlo et al., 2012; Vila et al., 2013, 2016; Spadari et al., 2019). MFS is an alkylphosphocholine compound initially investigated as an anticancer agent and currently recommended for the treatment of leishmaniasis (Dorlo et al., 2012; Almeida Pachioni et al., 2013). However, MFS has important side effects upon oral or systemic administration. The main side effects of oral MFS are related to its surfactant properties and include gastrointestinal discomfort; systemically, MFS has hemolytic and hepatic effects (Dorlo et al., 2012).

Incorporation into nanocarriers improves the pharmacological characteristics of antifungals such as AMB and MFS (da Gama Bitencourt et al., 2016; Spadari et al., 2017; Souza and Amaral, 2017), enabling prolonged drug release with reduced toxicity, administration frequency, dose and costs. Alginate-based nanocarriers can be used to encapsulate antifungals (azoles and polyenes) (Spadari et al., 2017), with the important advantage that alginate is non-toxic, non-immunogenic, biodegradable, biocompatible, and mucoadhesive (Cheng et al., 2012; Paques et al., 2014). Recently, our group showed that alginate nanoparticles can be used as MFS delivery systems, promoting sustained release and leading to a significant reduction of hemolytic effect and toxicity in a *Galleria mellonella* larval model (Spadari et al., 2019). Using this larval model, we also demonstrated the efficacy of miltefosine-loaded alginate nanoparticles (MFS-AN) for treatment of cryptococcosis and candidiasis (Spadari et al., 2019).

Microemulsions (ME) are thermodynamically stable lipid nanocarriers composed of droplets in the nanometer range (generally 10–100 nm); they improve drug efficacy and bioavailability, and can be used as carriers of lipophilic and hydrophilic molecules (Pepe et al., 2013; Thomas et al., 2014; Callender et al., 2017). Moreover, depending on composition, ME can undergo phase transformation forming liquid-crystalline gels *in vivo* upon uptake of vaginal fluids, providing sustained drug release at the site of administration (Phelps et al., 2011).

The aim of this study was to evaluate the antifungal efficacy of AMB and MFS in alginate- and lipid-based nanocarriers in a murine model of vaginal candidiasis. Here, the efficacy and administration frequency necessary for treatment with different topical formulations were assessed and compared with those of a conventional cream formulation currently used against human vaginal candidiasis.

## Materials and Methods

### Microorganisms

The reference strain *C. albicans* (SC5314) was stored in brain heart infusion broth (BHI, Becton, Dickinson and Company, United States) with 20% glycerol at −80°C and recovered in Sabouraud dextrose medium at 35°C, for 24–48 h. Yeasts were maintained on Sabouraud dextrose agar (SDA, Becton, Dickinson and Company, United States) at 4°C and subcultured in the same medium at least twice, at 35°C for 24 h, to obtain optimum fungal growth before assays.

### Drugs

Miltefosine (MFS, Cayman Chemical Company, United States) was diluted in sterile distilled water or propylene glycol and amphotericin B deoxycholate (AMB, Sigma-aldrich, United States) was diluted in dimethylsulfoxide (DMSO, Sigma-aldrich, United States). Both drugs were diluted prior to preparation of the formulations, as described below.

### Antifungal Susceptibility Testing

To confirm the minimum inhibitory concentration (MIC) values for AMB and MFS, the antifungal susceptibility testing was performed by the broth microdilution technique using the Clinical and Laboratory Standards Institute protocol M27 (CLSI, 2017).

### Vaginal Cream Formulations

The conventional vaginal cream formulations were prepared using 10% Polawax wax, 2% mineral oil, and 5% propylene glycol (in distilled water at pH 4.5) (de Freitas et al., 2018); containing either 1.25% amphotericin B (AMB-CR) or 2% miltefosine (MFS-CR).

### Microemulsion Formulations

A non-aqueous microemulsion (ME), in which water was replaced by propyleneglycol, was prepared as previously described (Carvalho et al., 2017), by mixing 20% propyleneglycol, 24.8% phosphatidylcholine, 5.6% tricaprilin, and 49.6% monoolein in a 37°C water bath until completely dissolved. Then, 1.25% AMB (AMB-ME) or 2% MFS (MFS-ME) were incorporated into the microemulsion. To confirm that the ME formulation would swell upon contact with the aqueous environment of the vagina, 100 mg ME were placed in contact with 500 μl of distilled water and incubated in a 37°C water bath for 48 h. The percentage of water absorbed by the formulation was determined as the difference between the weight before and after incubation with distilled water, and the types of liquid crystalline phases formed were assessed by polarized light microscopy, in a Leica DM2700P microscope equipped with a Leica DMC 2900 camera (Leica Microsystems, Germany).

### Alginate Nanoparticle Formulation

A formulation of MFS encapsulated in alginate nanoparticles (MFS-AN) was prepared by emulsification using the external gelation method, as previously described (Ishida et al., 2017; Spadari et al., 2019). Briefly, 3 mg MFS were dissolved in 1% (w/v) sodium alginate aqueous solution and mixed with 3% (w/v) sorbitan monooleate (SPAN 80) in sunflower oil to form an emulsion. Induction of nanoparticle formation was triggered by dripping 0.2 M CaCl_2_ with 0.5% polaxamer into the emulsion, under constant stirring. Then, the formulation was centrifuged and resuspended in 10% trehalose before freeze-drying, to obtain a fine powder (Ishida et al., 2017; Spadari et al., 2019). The mean size (Dz), polidispersion (Pdi), and zeta potential (ζ) of nanoparticles were measured in a Zetasizer NanoZS90 particle analyzer (Malvern Instruments, Worcestershire, United Kingdom). The lyophilized powder showed physical-chemical stability and *in vitro* antifungal activity for up to 30 days (Ishida et al., 2017; Spadari et al., 2019). For the *in vivo* assay MFS-AN (2% MFS) was incorporated in 1% (w/v) alginate gel.

### Animals

Female BALB/c mice (6–8 weeks old) were kept in pathogen-free conditions at the Laboratory of Animal Experimentation, Department of Microbiology, Institute of Biomedical Sciences, University of São Paulo (ICB, USP, São Paulo, Brazil). Food and water were provided *ad libitum* and all animals were treated according to the practices recommended by The National Institute of Health Animal Care guidelines (CONCEA, 2013). The experimental procedure proposed here (vaginal infection model and antifungal treatment) was reasoned according Hamad et al. (2004) and de Freitas et al. (2018) (Supplementary Figure S1), and was previously approved by the Ethics Committee on the Use of Animals (CEUA, ICB, USP; protocol no. 6439).

#### Vaginal Infection

The pseudoestrous phase was induced in female BALB/c mice by subcutaneous administration of 0.5 mg of 17-β-valerate-estradiol dissolved in 1 ml of sesame oil, 3 days before the establishment of vaginal infection (Hamad et al., 2004). Vaginal infection was established by intravaginal inoculation of 3 × 10^6^ *C. albicans* yeasts suspended in 10 μl of sterile PBS.

#### Treatment

Animals were divided into eight groups (*n* = 7 animals/group), and subjected, 24 h post-infection to one of the following treatments: MFS or AMB in a vaginal cream applied once a day for 6 days (MFS-CR(6×) and AMB-CR(6×) groups); MFS or AMB in microemulsion, administered every 2 days (three doses; AMB-ME(3×) and MFS-ME(3×) groups) or as a single dose (AMB-ME(1×) and MFS-ME(1×) groups); MFS in alginate nanoparticles, administered as a single dose (MFS-AN(1×) group); and an untreated group (Supplementary Figure S1). In addition, ME and AN groups (empty carriers) were included as controls. For each treatment dose, 30 μl of formulation were applied to the vaginal canal (∼0.64 mg of MFS in each formulation). On the 7th day post-infection, the animals were euthanized in a CO_2_ chamber and vaginal tissue was collected for mycological and histopathological analyses.

#### Mycological and Histopathological Analyses

For mycological analysis, five vaginas were excised, weighed, macerated and homogenized in 1 ml of sterile PBS. Serial dilutions (1:10) of the vaginal tissue homogenates were plated onto Sabouraud dextrose agar containing chloramphenicol (50 μg/ml) and incubated at 35°C for 48 h, and the number of colony forming units per gram of vaginal tissue (CFU/g) was determined by manual counting.

For histopathological analysis, the vaginas of two animals were excised, fixed with 10% formaldehyde in PBS and processed for conventional histological analysis using hematoxylin and eosin staining. Samples were analyzed by light microscopy in a Leica DM750 light microscope (Leica Microsystems, Germany), at 400× magnification. The fungal load was determined semi-quantitatively according to the scale: 0, no fungal load; + 1, up to five fungal elements per section; +2, ≥6 fungal elements per section; + 3, from 6 to 50 fungal elements per field; + 4, more than 50 fungal elements per field (Quintella et al., 2011).

#### *In vivo* Imaging System

The fluorescent dye Alexa Fluor^®^ 647 (Catalog no. A20106, Molecular Probes, United States) was used to evaluate the persistence of formulations in the vaginal canal, by whole body *in vivo* bioimaging. The following groups were included in these experiments: vaginal cream with Alexa Fluor 647 (CR + AF), microemulsion with or without Alexa Fluor 647 (ME + AF and ME groups, respectively), and alginate nanoparticles with or without Alexa Fluor 647 (AN + AF and AN groups, respectively) (*n* = 3 animals/group). The production of nanocarriers containing Alexa Fluor was performed as described in the previous sections wherein the fluorescent dye was added after production of formulation (vaginal cream and microemulsion) or it was dissolved in 1% (w/v) sodium alginate aqueous solution for incorporation in the alginate nanoparticles. Alexa fluor 647 is not expected to chemically interact/react with the nanocarriers, but to dissolve in the aqueous phase (Carvalho et al., 2019). The fluorescent dye was chosen for this assay because is a hydrophilic compound, that can be dissolved and incorporated in the nanocarriers, and its fluorescent signal do not interfere the auto-fluorescence from hair follicles and other skin structures of mice (Migotto et al., 2018).

Mice were anesthetized with isoflurane (Cristália, São Paulo, Brazil) and 30 μl of each formulation were administered intravaginally. Whole body images of treated animals were obtained using the IVIS Spectrum bioimaging system (PerkinElmer Life Sciences, Waltham, MA, United States) to detect the presence of formulations by dye fluorescence emission in the vaginal canal. Images were captured at 0, 1, 2, 3, 6, 7 days post-administration, and the fluorescence intensity was measured. The following instrument settings were used for comparison among different groups: exposure time 5 s, binning factor eight, excitation/emission 465/540 nm (Migotto et al., 2018).

### Statistical Analysis

Statistical analysis was performed by one-way ANOVA followed by Dunnett’s test, using the Graphpad Prism 5.0 program (GraphPad, La Jolla, CA, United States), with a 95% confidence interval.

## Results and Discussion

Fungal vaginitis, particularly VVC and RVVC represent important public health problems given their debilitating and long term effects on women’s quality of life, including both physical and mental symptoms such as anxiety and depression, in chronic and recurrent cases (Sobel, 2016; Denning et al., 2018). The recurrent condition also decreases women’s productivity, leading to an estimated productivity loss of up to U$$ 14.39 billion annually (Denning et al., 2018). Current therapies are often ineffective against VVC and RVVC, with particular need for safe and effective topical treatments to improve women care.

Both AMB and MFS have broad spectrum of antifungal activity, fungicidal and post-antifungal effects (Dorlo et al., 2012; Spadari et al., 2018). Importantly, these drugs are effective against *Candida* spp. strains resistant to azoles, and also against biofilms, which are associated with disease persistence and with increased severity of vaginal fungal infections (Muzny and Schwebke, 2015; Vila et al., 2013, 2016; Spadari et al., 2019). Initially, we confirmed the antifungal activity of MFS and AMB on the *C. albicans* reference strain SC5314 using the assay described in the M27 document (CLSI, 2017), with MIC values of 1 μg/ml and 0.12 μg/ml (for MFS and AMB, respectively), which are in agreement with values reported previously (Vila et al., 2016; Spadari et al., 2019).

As AMB is an important antifungal agent since 1956, several *in vivo* studies have shown the efficacy of different formulations for AMB using animal models of invasive or cutaneous fungal infections and it is used actually to treat many candidiasis clinical forms (Pappas et al., 2015). In contrast, studies *in vivo* for MFS antifungal activity is still insufficient. Previous studies reported a reduction in *Cryptococcus* dissemination in the central nervous system using murine model upon treatment with 3.6 mg/kg MFS (Widmer et al., 2006); and the pre-treatment of mouse oral mucosa with an aqueous solution containing 2 mg/ml MFS decreased *Candida* infection (Vila et al., 2015). Here, we evaluated the antifungal efficacy of MFS or AMB in two nanocarriers (microemulsion and alginate nanoparticles) and compared with conventional formulation testing on vaginal candidiasis model.

In our model, we chose to treat vaginal candidiasis by the intravaginal route of administration, because local action formulations can contain high doses of active compounds without causing significant systemic side effects, by avoiding gastrointestinal contact and the “first pass” effect in the liver (Bachhav and Patravale, 2009; Leyva-Gómez et al., 2018). The pharmaceutical forms commonly used in the topical route include creams, gels, vaginal ovules, capsules, tablets, among others; despite this variety, the major disadvantage is the fact that drug release is not prolonged and low retention to the vaginal epithelium, requiring prolonged duration of therapy and/or higher frequency of administered doses (Bachhav and Patravale, 2009; Leyva-Gómez et al., 2018). Thus, we tested here the use of nanocarriers (microemulsions and alginate nanoparticles), in an attempt to overcome the lack of persistence in intravaginal treatment using conventional formulations.

The lipid microemulsion (ME) was developed using components generally regarded as safe and used in other vaginal/rectal formulations (Ganem-Quintanar et al., 2000; Garg et al., 2001). Previous studies demonstrated that vaginal administration of ME composed of phosphatidylcholine and/or monoolein (as employed here) caused no local histological changes such as epithelial thickening or infiltration of inflammatory cells (Refai et al., 2017; Aboud et al., 2018; Wang et al., 2019); and no decreases in cell viability was observed when the ME was used up to 10 mg/ml assessed in tumor and non-transformed epithelial cells in 2D cultures *in vitro* (Lopes et al., 2019). For comparison, other ME containing larger amounts of non-ionic surfactants caused no significant reduction on the viability of fibroblasts when used at 10–50 μg/ml (Hosmer et al., 2009; Phelps et al., 2011), indicating that the ME developed here should be safer.

**FIGURE 1:**
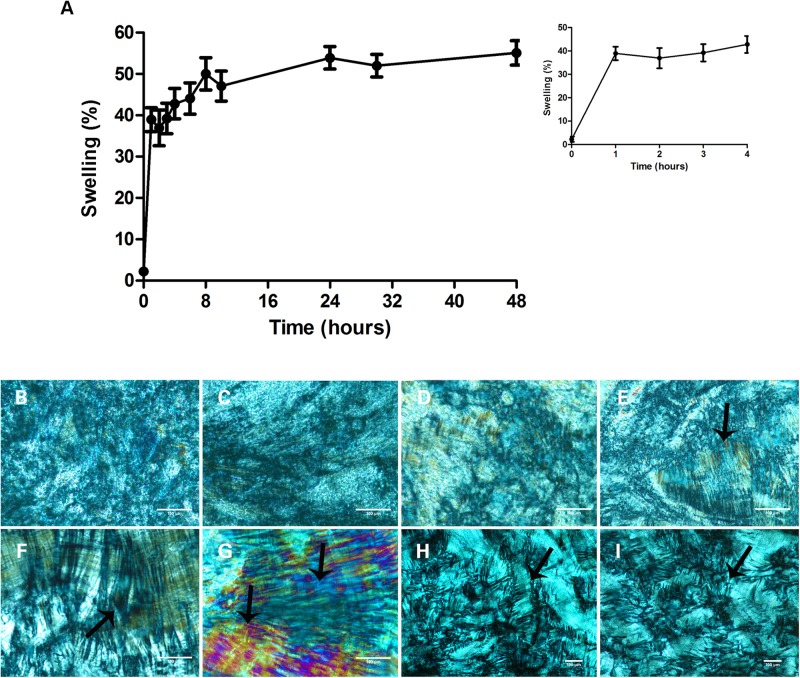
Microemulsion (ME) swelling after contact with an aqueous environment. **(A)** Percentage of microemulsion swelling during 48 h of contact with water, at 37°C bath. The inset in the **(A)** is an enlarged figure in the times ranging from 1 to 4 h of incubation. Data represent mean ± SD values of three independent experiments. **(B–I)** Microemulsion morphology as seen by polarized light microscopy at the following incubation times: 1 h **(B)**, 2 h **(C)**, 3 h **(D)**, 4 h **(E)**, 6 h **(F)**, 24 h **(G)**, and 48 h **(H,I)**. The hexagonal phase, characterized by the presence of two-dimensional structure containing long cylindrical arrangements (arrows), indicates the formation of a liquid-crystalline gel capable of sustained drug delivery. The hexagonal phase is observed after 4 h of incubation with water **(E)**, and is maintained for 48 h **(H,I)**. Scale bars = 100 μm.

ME swelling and transition to gel *in vivo* is important to prolong drug release (Carvalho et al., 2017; Phelps et al., 2011). Thus, we verified if the ME used here would swell upon contact with an aqueous environment and undergo phase transition to gel. ME swelling was observed within the first hour of incubation with distilled water at 37°C and remained constant for 48 h (Figure 1A), generating a hexagonal phase gel after 4 h of contact with water (Figure 1E) unlike previous hours (Figures 1B–D). This “mesophase” can be described as a two-dimensional structure containing long cylindrical arrangements as described previously by Lopes et al. (2006), and was maintained for 48 h in contact with water (Figures 1E–I, black arrows). Moreover, the transformation of the liquid-crystalline gels *in vivo* upon uptake of vaginal fluids as well as ME non-toxic profile on mucosa and skin (Phelps et al., 2011; Carvalho et al., 2017) are both important characteristics for topical application used here.

The natural polymer alginate has been explored in the last decade for development of drug delivery systems, including antifungals as azoles and polyenes, due to its non-toxicity and non-immunogenic properties when systemically or topically administered (reviewed by Spadari et al., 2017). In previous studies, we demonstrated that alginate nanoparticles promoted sustained release of MFS (∼ 8% or ∼125 μg/ml) in aqueous medium for up to 24 h (Ishida et al., 2017; Spadari et al., 2019). Furthermore, MFS encapsulated in alginate nanoparticles showed reduced toxicity compared with free MFS, and antifungal efficacy against candidiasis and cryptococcosis, in an invertebrate model (Spadari et al., 2019). Thus, we tested MFS-loaded alginate nanoparticles (MFS-AN) as an option for antifungal therapy in murine vaginitis. The MFS-AN used in this study exhibited mean size of 280 – 350 nm, polydispersity < 0.3 (indicating low polydispersity) and negative zeta potential of approximately – 25 mV, in agreement with previous results from our group (Ishida et al., 2017; Spadari et al., 2019).

Both nanocarriers containing AMB or MFS were evaluated in murine model of vaginal candidiasis. Female BALB/c mice were infected with *C. albicans* SC 5314 (3 × 10^6^ yeasts) intravaginally due to its pathogenicity previously described in oral/vaginal mucosa (Wang et al., 2016); and this model could properly mimic complicated clinical conditions and provides a valuable means for antifungal assay *in vivo* (Wang et al., 2016). After 24 h of fungal infection, the mice were treated with AMB or MFS in microemulsions or alginate nanoparticles and compared with a conventional formulation (vaginal cream). In addition, we tested two different treatment schedules for the microemulsion treatments (“3×” and “1×,” for repeated dosing every 2 days or a single dose, respectively) and only single dose for alginate nanoparticles.

**FIGURE 2:**
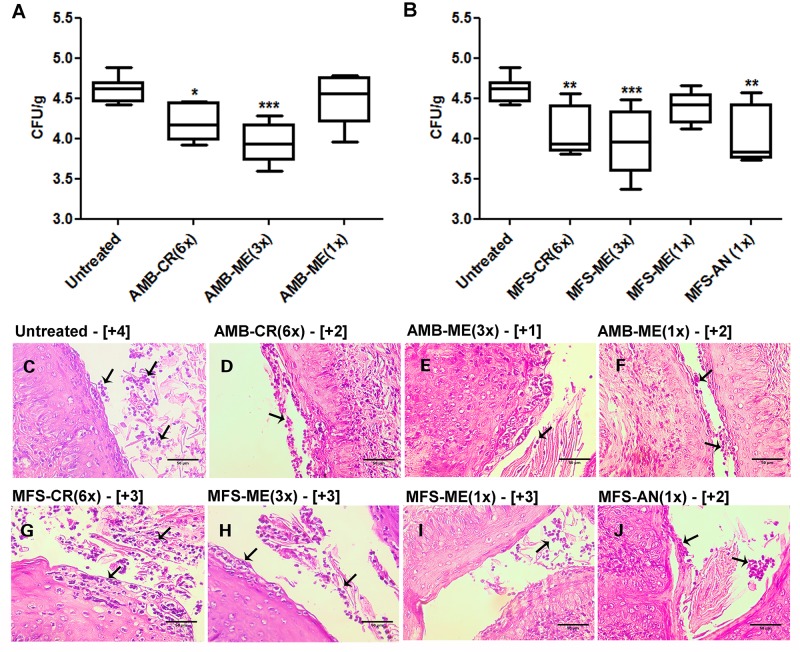
Fungal burden of *Candida albicans* in vaginal tissue of BALB/c mice after treatment with formulations contained amphotericin B (AMB) or miltefosine (MFS) in a vaginal cream (AMB-CR and MFS-CR), in microemulsion (AMB-ME and MFS-ME) or encapsulated in alginate nanoparticles (MFS-AN). The numbers in parenthesis indicate the number of doses administered. **(A,B)** Fungal burden quantification (by colony forming units/g – CFU/g) using a plaque assay (*n* = 5 animals/group). The amplitude of the bars is defined by the maximum and minimum values. ^∗^*p* < 0.05, ^∗∗^*p* < 0.01 and ^∗∗∗^*p* < 0.001 when compared with the untreated group (one-way ANOVA, followed by Dunnett’s test). **(C–J)** Hematoxylin-eosin stained histopathological sections of vaginal tissue kept untreated or treated with AMB or MFS in different formulations. Arrows indicate the fungal cells. **(C)**: untreated group; **(D)**: AMB-CR (6×); **(E)**: AMB-ME (3×); **(F)**: AMB-ME (1×); **(G)**: MFS-CR (6×); **(H)**: MFS-ME (3×); **(I)**: MFS-ME (1×); and **(J)**: MFS-AN (1×). Results of semi-quantitative fungal load analysis from vaginal tissue sections are indicated in brackets (*n* = 2 animals/group). Scale bars = 50 μm.

Among AMB formulations, AMB-CR(6×) and AMB-ME(3×) significantly reduced the fungal burden in the vaginal tissue, but not as a single dose of AMB-ME, which did not control fungal infection (Figure 2A). We observed similar results for treatment with MFS-CR(6×) and MFS-ME(3×) decreasing the fungal burden significantly compared with the untreated group, while a single dose of MFS-ME (1×) could not control the infection (Figure 2B). In addition, we observed a significant reduction in fungal burden after vaginal candidiasis treatment using a single dose of MFS-AN, and the efficacy of this treatment was comparable with that of the other effective formulations (Figure 2B). The semi-quantitative analysis of fungal load in vaginal tissue based on histological section examination (Figures 2C–J) corroborated the fungal burden data (Figures 2A,B), indicating that the treatments with AMB-ME(3×), MFS-ME(3×), and MFS-AN(1×) were more expressive, because they combined efficacy (similar or higher than that of conventional vaginal cream) with the possibility to reduce administration frequency. In addition, we also evaluated if the nanocarriers *per se* could interfere on the antifungal activity of formulations; and the CFU/g data were similar to the untreated group (data not show). Similar result was observed previously for vaginal cream using the same vaginal candidiasis model (de Freitas et al., 2018).

Amphotericin B and MFS incorporated into microemulsions were also effective in the control of vaginal candidiasis when used in three doses, and both antifungals presented a similar effect. The advantage of the microemulsion used here compared to the alginate-based nanoparticle is the *in situ* (i.e., in the vaginal mucosa) formation of a liquid crystalline system with a gel-like appearance upon contact with water (Geraghty et al., 1996; Lopes et al., 2006; Phelps et al., 2011). The gel formed after ME swelling is more viscous, which improves the residence time in the vaginal canal (Geraghty et al., 1997; Rodero et al., 2018; Jie et al., 2019). Advantages resulting from this property include the lower probability of leaking after delivery and the possibility of reducing the dosing frequency compared to less viscous systems (Rodero et al., 2018; Jie et al., 2019). Thus, the fluid ME can be considered a ready-to-use delivery system and does not need to be incorporated in a gel or in any other dosage form for administration since it spontaneously forms a gel *in situ*. On the contrary, alginate nanoparticles had to be incorporated in a gel for its topical application, which requires fine-tuning its viscosity for an easy administration.

**FIGURE 3:**
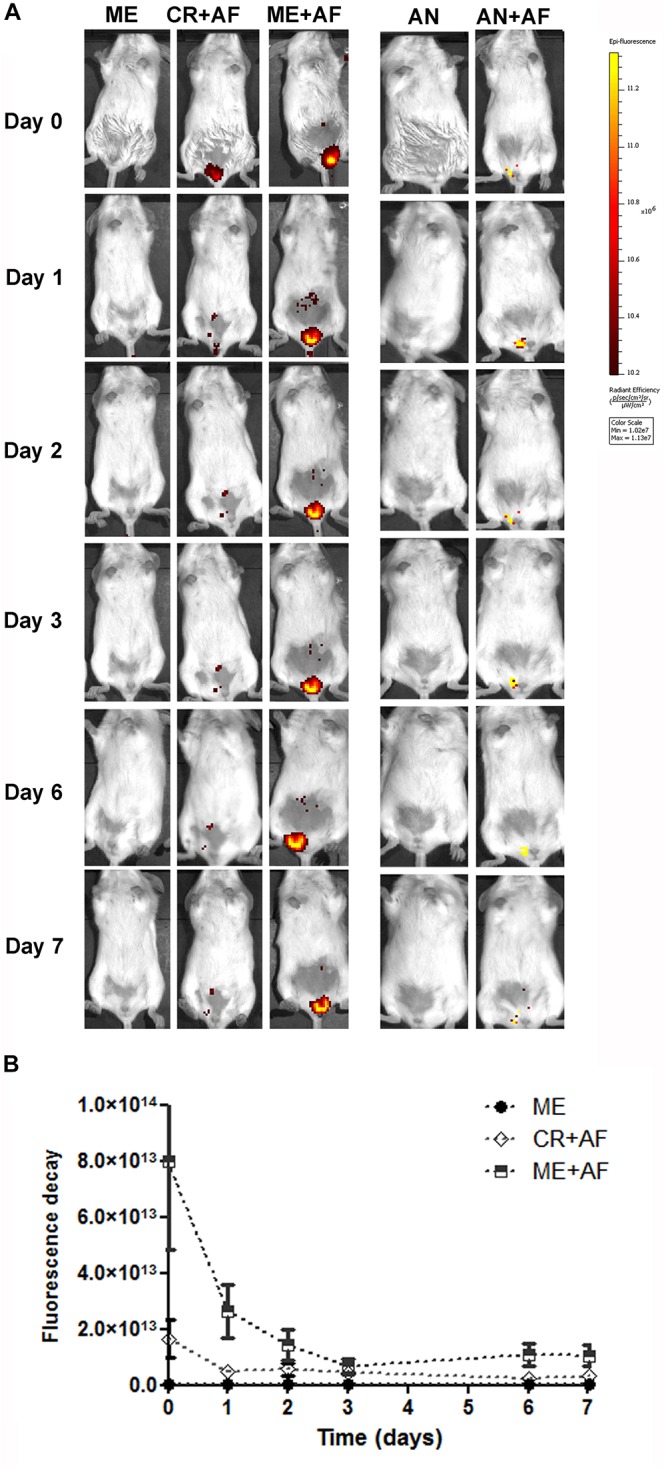
*In vivo* imaging of BALB/c female mice treated intravaginally with different drug nanocarriers labeled with Alexa Fluor 647. **(A)** Images at different time points after intravaginal administration of microemulsion with or without Alexa Fluor 647 (ME + AF and ME, respectively), vaginal cream with Alexa Fluor 647 (CR + AF) and alginate nanoparticles with or without Alexa Fluor 647 (AN + AF and AN). Colors indicate fluorescence intensity according to the scale on the right. **(B)** Fluorescence decay after administration of Alexa Fluor 647 labeled nanocarriers (*n* = 3 animals/group; values represent the fluorescence intensity median from 10 images). The fluorescence intensity in CR + AF and ME + AF was significantly above background (*p* < 0.01 compared with ME), and the fluorescence intensity was significantly higher and more prolonged in the ME + AF group, compared with the CR + AF group (*p* < 0.05), showing that microemulsion persists in the vaginal canal throughout the evaluation period (7 days).

To evaluate if the nanocarriers would remain longer in the vaginal canal compared to a conventional cream, we treated animals with formulations containing a fluorescent dye (Alexa Fluor 647) and assessed if the fluorescence signal persisted longer in the vaginal canal using *in vivo* imaging. Our data showed the persistence of the fluorescence signal in the vaginal canal for the duration of the treatment period using lipid- and alginate-based nanocarriers systems (Figure 3). The microemulsion (ME + AF) remained in the vaginal canal for up to 7 days (Day 7), and the fluorescence intensity was significantly higher than that obtained with the vaginal cream used as conventional formulation (CR + AF) (*p* < 0.05), which was barely detectable in the vaginal canal after 1-day post-administration (Day 1) (Figures 3A,B).

The administration of alginate nanoparticles also resulted in fluorescence signals that persisted for up to 7 days in the vaginal canal (Day 7) (Figure 3A); however, the Alexa Fluor 647 was not fully incorporated into the alginate nanoparticles, as evidenced by the lower fluorescence intensity of AN + AF right after administration (Day 0) (Figure 3A). Nevertheless, we still detected considerable AN + AF fluorescence at 7 days post-administration (Day 7), suggesting that the particles provided prolonged release of the encapsulated probe (Figure 3A). In addition to the fact that alginate is a polymer with strong mucoadhesive properties, we reported previously that MFS is released in a prolonged fashion when encapsulated in alginate nanoparticles (Ishida et al., 2017; Spadari et al., 2019); together, these two properties of alginate nanoparticles might explain the sustained antifungal effect of MFS in the MFS-AN formulation, observed here in the treatment of murine vaginal candidiasis.

MFS-AN was the best performing formulation overall, given its strong effect as a single-dose treatment. The reason for the higher antifungal efficacy of MFS-AN formulation compared to the microemulsion is unclear. One possibility is the difference in the drug release profile. However, we were not able to compare drug release from the formulations due to methodological difficulties; both drugs AMB and MFS aggregate in water-based solvents, hindering drug transport across semi-permeable membranes (employed to separate the nanocarrier from the bulk release medium), and underestimating release (Barioni et al., 2015). Further studies for method adaptation are necessary to address this issue. Nevertheless, the data presented here demonstrated that MFS is active *in vivo* against vaginal infection by *Candida* in three different formulations.

In conclusion, our results demonstrated that lipid- or alginate-based nanocarrier formulations of AMB and MFS provide sustained antifungal activity after intravaginal application and may represent an important alternative for effective topical treatment of vaginal candidiasis. Compared with the standard treatment with vaginal cream, alginate nanoparticles and microemulsions reduced the number of antifungal doses required to decrease the fungal burden in infected tissues. These formulations could contribute to improve treatment and, thus, the quality of life of women diagnosed with VVC and RVVC.

## Data Availability Statement

All datasets generated for this study are included in the article/Supplementary  Material.

## Ethics Statement

The animal study was reviewed and approved by Ethics Committee on the Use of Animals (CEUA, ICB, USP; protocol no. 6439).

## Author Contributions

FB designed and performed all experiments, analyzed the results, and drafted the manuscript. JM standardized the microemulsion formulation. CS standardized the protocol for the production of alginate nanoparticles and contributed to the *in vivo* assays. GS contributed with the *in vivo* imaging experiments. KI and LL designed and supervised all experiments, analyzed the data, and wrote the manuscript. All authors gave final approval of the version to be published and agreed to be accountable for all aspects of the work.

## Conflict of Interest

The authors declare that the research was conducted in the absence of any commercial or financial relationships that could be construed as a potential conflict of interest.
