# Supplementary material for: Nanocarriers Provide Sustained Antifungal Activity for Amphotericin B and Miltefosine in the Topical Treatment of Murine Vaginal Candidiasis
Source: Front Microbiol. 2020 Jan 10;10:2976. doi: 10.3389/fmicb.2019.02976 (PMC6965356; doi:10.3389/fmicb.2019.02976)
Supplement: Supplementary file 1 [file Data_Sheet_1.docx]

**Supplementary material**


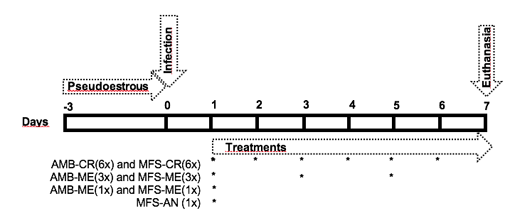


**FIG S1** Treatment schedules tested against murine vaginal candidiasis of formulations containing amphotericin B (AMB) or miltefosine (MFS) in a vaginal cream (AMB-CR and MFS-CR), in microemulsion (AMB-ME and MFS-ME) or encapsulated in alginate nanoparticles (MFS-AN). The pseudoestrous phase was induced with 17-β-valerate-estradiol, 3 days before intravaginal infection with *Candida albicans* SC5314 (3 x 10^6^ yeasts/10 µL), and treatments were started 24 h post-infection. Intravaginal treatment administrations are indicated by an asterisks (*). Animals were euthanized on the 7^th^ day post-infection and the vaginal tissue collected for mycological and histopathological analyses.
